# Flourine

**Published:** 1844-11

**Authors:** 


*Flourine.*—Drs. Will and Fresenius have detected flourine in the ashes of plants—it exists in all the cereals. It has been found in the bones of all recent animals thus far examined, also in fosil bones.—*Ibid.*

